# Ameliorative Effects of PACAP against Cartilage Degeneration. Morphological, Immunohistochemical and Biochemical Evidence from *in Vivo* and *in Vitro* Models of Rat Osteoarthritis

**DOI:** 10.3390/ijms16035922

**Published:** 2015-03-13

**Authors:** Salvatore Giunta, Alessandro Castorina, Rubina Marzagalli, Marta Anna Szychlinska, Karin Pichler, Ali Mobasheri, Giuseppe Musumeci

**Affiliations:** 1 Department of Biomedical and Biotechnological Sciences, Human Anatomy and Histology Section, School of Medicine, University of Catania, Via S. Sofia 87, 95123 Catania, Italy; 2 Department of Pediatrics, Clinic for Pediatrics I Medical University of Innsbruck, Anichstr. 35, A-6020 Innsbruck, Austria; 3 The D-BOARD European Consortium for Biomarker Discovery, Department of Veterinary Preclinical Sciences, School of Veterinary Medicine, Faculty of Health and Medical Sciences, University of Surrey, Guildford GU2 7XH, UK; 4 Arthritis Research UK Centre for Sport, Exercise and Osteoarthritis, Arthritis Research UK Pain Centre, Medical Research Council and Arthritis Research UK Centre for Musculoskeletal Ageing Research, University of Nottingham, Queen’s Medical Centre, Nottingham NG7 2UH, UK; 5 Center of Excellence in Genomic Medicine Research (CEGMR), King Fahd Medical Research Center (KFMRC), King AbdulAziz University, Jeddah 21589, Saudi Arabia

**Keywords:** pituitary adenylate cyclase-activating polypeptide (PACAP), Osteoarthritis, anterior cruciate ligament transection (ACLT), immunohistochemistry, Bcl-2, BAX, caspase-3

## Abstract

Osteoarthritis (OA); the most common form of degenerative joint disease, is associated with variations in pro-inflammatory growth factor levels, inflammation and hypocellularity resulting from chondrocyte apoptosis. Pituitary adenylate cyclase-activating polypeptide (PACAP) is a neuropeptide endowed with a range of trophic effects in several cell types; including chondrocytes. However; its role in OA has not been studied. To address this issue, we investigated whether PACAP expression is affected in OA cartilage obtained from experimentally-induced OA rat models, and then studied the effects of PACAP in isolated chondrocytes exposed to IL-1β *in vitro* to mimic the inflammatory milieu of OA cartilage. OA induction was established by histomorphometric and histochemical analyses. Changes in PACAP distribution in cartilage, or its concentration in synovial fluid (SF), were assessed by immunohistochemistry and ELISA. Results showed that PACAP abundance in cartilage tissue and SF was high in healthy controls. OA induction decreased PACAP levels both in affected cartilage and SF. *In* *vitro*, PACAP prevented IL-1β-induced chondrocyte apoptosis, as determined by MTT assay; Hoechst staining and western blots of apoptotic-related proteins. These changes were also accompanied by decreased i-NOS and COX-2 levels, suggesting an anti-inflammatory effect. Altogether, these findings support a potential role for PACAP as a chondroprotective agent for the treatment of OA.

## 1. Introduction

Osteoarthritis (OA) is the most common type of arthritic disease [1]. In OA articular cartilage is lost through a combination of degenerative and inflammatory phenomena, which are often accompanied by joint swelling, pain, stiffness (crepitus) and loss of joint mobility [2,3]. The pathogenesis of OA is multifactorial and is considered to be a metabolically active process, predominantly due to the poor regenerative properties of articular cartilage after damage and traumatic injury [4]. The root causes of OA are still unknown, but metabolic, genetic, chemical, inflammatory mediators and mechanical stress are potential causative factors that may play important roles in its development [5]. Numerous studies have reported that chondrocytes from patients with OA show increased levels of reactive oxygen species (ROS) within the joint microenvironment, that in turn may be the leading cause of structural and functional alterations in the extracellular matrix (ECM) of cartilage [6,7]. Increased oxidative stress caused by excessive ROS production has also been shown to promote the activation of inflammatory mechanisms [8], as well as determining variations in the pro-inflammatory growth factor levels [9,10], which in turn have been associated with the promotion of apoptotic death of chondrocytes in OA [11]. Although the downstream targets of ROS-induced cellular responses have been extensively studied in chondrocytes and synoviocytes [12,13,14], only a few pharmacological compounds have been identified as potentially useful antioxidant agents [15]. 

It has been widely reported that chondrocytes have the capacity to produce a variety of cytokines and inflammatory mediators, which have been demonstrated to play a pivotal role in the development and progression of OA. In particular, IL-1β seems prominent to cartilage destruction [16,17]. The latter mimics a number of tissues and molecular responses seen in OA, such as reduction of chondrocytes viability, up-regulation of pro-apoptotic proteins, increase of catabolic enzymes activity and activation of signaling pathways leading to up-regulation of the inflammatory gene products [18,19].

Interestingly, a recent study reported that isolated chondrocyte cultures express functional pituitary adenylate cyclase-activating polypeptide (PACAP) receptors, suggesting a potential chondroprotective role for this peptide in experimentally induced oxidative stress [20,21] and inflammatory conditions.

PACAP is a member of the vasoactive intestinal polypeptide (VIP)/secretin/glucagon peptide superfamily that was originally isolated from extracts of rat hypothalamus [22]. The protective effect of PACAP has been demonstrated against several types of insults, including oxidative stress, ischemic insults or mechanical trauma [23,24,25]. The anti-inflammatory action of this peptide has also been reported; indeed, it appears to inhibit the expression and release of proinflammatory cytokines and chemokines, and enhances the production of the anti-inflammatory factors in several conditions [26]. Furthermore, data reported that systemic treatment with PACAP proved to greatly reduce the clinical symptoms and alter the pathogenic and cytokine profiles in animal models of some autoimmune diseases such as rheumatoid arthritis, Crohn’s disease, septic shock, and multiple sclerosis [27]. In spite of the mentioned data, recently, Botz and coauthors reported that in PACAP knockout mice with serum transfer arthritis, inflammatory cell accumulation is reduced in early phases of the disease while in the later phases, immune cell function appears to be increased [28]. The protective effect of PACAP in autoimmune diseases is attributed to its capacity to act as both activator and inhibitor of T helper cells. Indeed, this peptide has been shown to be able to promote Th2-type, and inhibit Th1-type responses *in vivo* and *in vitro*, through several mechanisms, including preferential survival of Th2 effectors and subsequent generation of Th2 memory cells [29].

PACAP binds with high affinity to three specific G protein–coupled receptors classified into two subtypes known as PAC1 (with at least 8 different isoforms) and VPAC types (including VPAC1 and VPAC2 subtypes). VPAC type receptors show similar affinity to PACAP and its homolog vasoactive intestinal polypeptide (VIP), whereas PAC1 types bind PACAP with 100- to 1000-fold higher affinity than VIP. Expression studies have shown that PACAP and its related receptors are localized to various peripheral organs, such as gonads [30], intestinal tract [31] and urinary system [32], and the presence of PACAP has also been verified in human milk and blood plasma [33]. However, it has not been determined whether PACAP is also present in synovial fluid (SF) and its potential trophic activity on cultured chondrocytes has never been studied in detail.

In the present study, we addressed these issues by performing comparative analyses of PACAP expression/distribution in healthy cartilage tissue and in degenerate cartilage from an *in vivo* rat model of OA. In addition, we also evaluated whether the concentration of PACAP in SF is altered by induction of OA. Furthermore, since local inflammation leads to loss of chondrocytes, we also performed additional studies in isolated chondrocyte cultures *in vitro,* in the attempt to appraise the effects of PACAP on cell viability and inflammation after exposure to increasing concentrations of the pro-inflammatory cytokine interleukin-1 β (IL-1β). Our data demonstrated that: (1) PACAP is expressed at moderate/high levels in the articular cartilage, so is its concentration in the SF; (2) experimental OA remarkably dampened peptide levels in cartilage tissue and SF and this inversely correlated with IL-1β concentration in the SF; (3) PACAP inhibited IL-1β-induced apoptosis, as well as the expression of i-NOS and COX-2 in cultures of isolated chondrocytes. Taken together, the present findings support the hypothesis that PACAP may be a potentially suitable biological agent for the treatment of degenerative/inflammatory diseases of joints such as OA and other related osteoarticular disorders.

## 2. Results and Discussion

### 2.1. Histomorphometric Analyses

**Figure 1:**
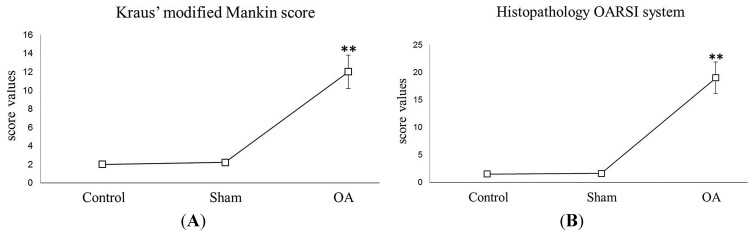
**Graph A**, Kraus’ modified Mankin score. Kraus’ modified Mankin score among groups. Results are presented as the mean ± SEM. One-way ANOVA followed by Tukey’s *post-hoc* test was used to evaluate statistical significance of the results. ** *p* < 0.01 when compared to the control groups; **Graph B**, Histopathology OARSI system. Histopathology OARSI system among groups. Results are presented as the mean ± SEM. One-way ANOVA followed by Tukey’s *post-hoc* test was used to evaluate statistical significance of the results. ** *p* < 0.01 when compared to the control groups.

The histomorphometric parameters, performed in control and in sham groups ((without anterior cruciate ligament transection (ACLT)), confirmed that the animals demonstrated no sign of cartilage degeneration with an intact and normal cartilage structure, whilst in the OA group (with ACLT) the animals demonstrated evidence of pathological changes to cartilage and severe OA; in fact horizontal cleavage tears or flaps and deep lesions were present. Examination of the OA group confirmed the development of degenerative processes in articular cartilage, which were significantly different from the control groups, as confirmed by Kraus’ modified Mankin score (Figure 1A), and histopathology “Osteoarthritis Research Society International” (OARSI) system score (Figure 1B). The inter-observer variability among three observers for the MANKIN system showed a similar good intra-class correlation coefficient (ICC > 0.80) as for the OARSI system (ICC > 0.70). Repeated scoring by investigators showed very good agreement (ICC > 0.90).

### 2.2. Histology and Histochemistry

**Figure 2:**
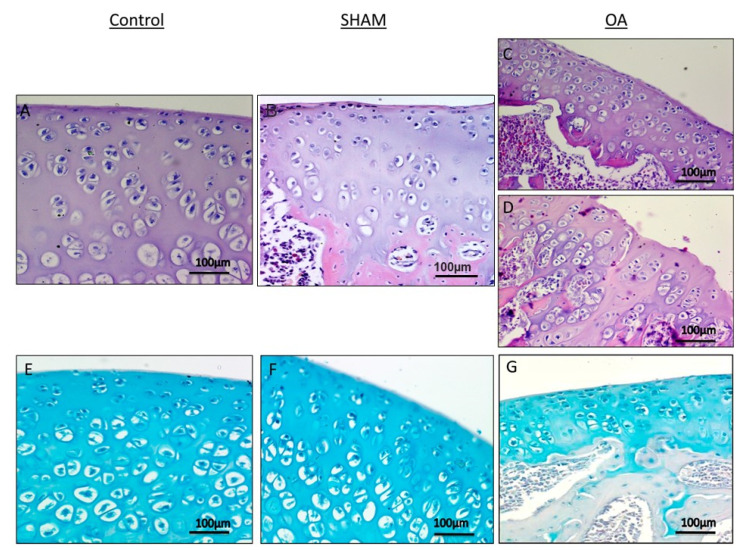
Histological and histochemical evaluation. (**A**,**B**) Histology (H&E staining) demonstrated the absence of structural alterations in control groups (without anterior cruciate ligament transection (ACLT)). In the superficial zone, cells appear flat and small; in the middle and deep zone, cells are organized in columns. Magnification ×20; Scale bars: 100 µm; (**C**) Histology (H&E staining) demonstrated evidence of structural alterations in cartilage with moderate signs of OA (with ACLT). The structural alterations included a reduction of cartilage thickness in the superficial and the middle zones. The tidemark is no longer intact and the subchondral bone shows fibrillation. Magnification ×20; Scale bars: 100 µm; (**D**) Histology (H&E staining) demonstrated signs of structural alterations in severe Osteoarthritis (OA) (with ACLT). Severe OA cartilage shows deep surface clefts, disappearance of cells from the superficial zone, cloning, and a lack of cells in the intermediate and deep zone, which are not arranged in columns. The cartilage layers (superficial zone, middle and deep zone) are completely absent. Magnification ×20; Scale bars: 100 µm; (**E**,**F**) Histochemistry (toluidine blue staining) showed an absence of structural alterations and preserved GAG, in control groups (without ACLT), as indicated by the intense toluidine blue staining. Magnification ×20; Scale bars: 100 µm; (**G**) Histochemistry (toluidine blue staining) demonstrated signs of structural alterations in moderate and severe OA cartilage and loss of proteoglycans as evidenced by poor GAG preservation in the OA group (with ACLT), showing reduced toluidine blue staining. Magnification ×20; Scale bars: 100 µm.

Histology (H&E staining) and histochemistry (toluidine blue staining) demonstrated the absence of structural alterations in control and in sham groups (Figure 2A,B,E,F), while showing structural alterations, in the OA group (Figure 2C,D,G). The histological (H&E staining) analysis of cartilage from control groups (without ACLT), showed a well-preserved morphological structure (Figure 2A,B) and an intense toluidine blue staining (Figure 2E,F). In contrast to the OA group (with ACLT), where moderate structural alterations in OA cartilage included a reduction of cartilage thickness in the superficial and the middle zones (Figure 2C). The structure of the collagen network was damaged, thereby leading to thinning of the cartilage. In severe OA, the cartilage demonstrated deep surface clefts, disappearance of cells from the tangential zone, chondrocyte cloning, and loss of cells in the intermediate and radial zone, which are not arranged in columns. The tidemark is no longer intact and the subchondral bone shows signs of remodeling fibrillation (Figure 2D). The toluidine blue staining was also reduced, thus indicating loss of proteoglycans and reduction of GAG content (Figure 2G). Moreover, while the surface of healthy hyaline cartilage appeared white, shiny, elastic and firm, in OA the surface was dull and irregular.

### 2.3. Immunohistochemical Observations

**Figure 3:**
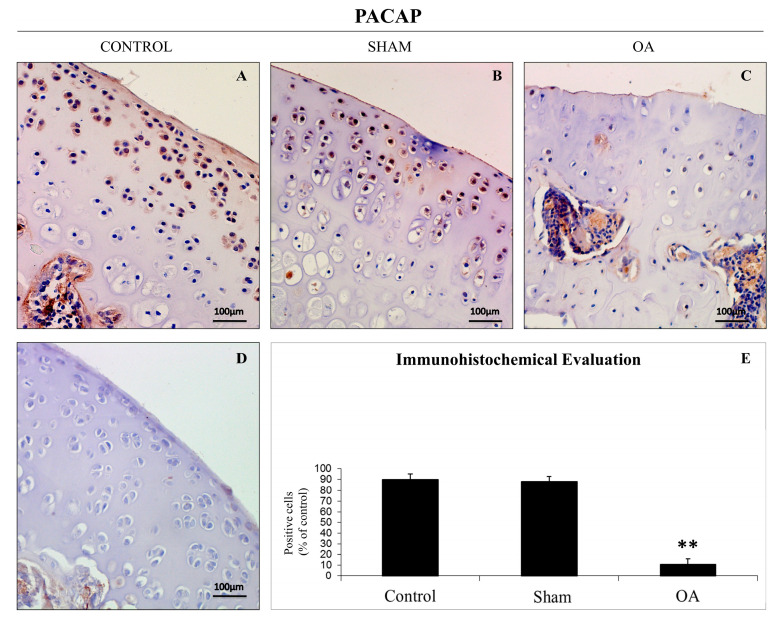
Pituitary adenylate cyclase-activating polypeptide (PACAP) immunoreactivity in healthy and OA cartilage tissue. (**A**,**B**) PACAP immunohistochemistry in control cartilage (without ACLT) exhibited a very strong (ES = ++++; IS = 4) immunostaining in chondrocytes from rat femoral articular cartilage (superficial and middle zone). Magnification ×20; Scale bars: 100 µm; (**C**) PACAP immunohistochemistry in moderate/severe OA cartilage (with ACLT) exhibited a weak/absent (ES = +; IS = 1) immunostaining in chondrocytes from rat femoral articular cartilage (superficial and middle zone). Magnifications ×20; Scale bars: 100 µm; (**D**) No immunostaining was observed in the negative control (ES = 0; IS = 0) treated with PBS in place of the primary antibody. Magnifications ×20; Scale bars: 100 µm; (**E**) Immunohistochemistry: percentage of PACAP positive cells out of the total number of cells counted in control groups and in OA group. Results are presented as the mean ± SEM. One-way ANOVA followed by Tukey’s *post-hoc* test was applied to evaluate the statistical significance of the results. ** *p* < 0.01 *vs.* control groups.

PACAP expression was evaluated by immunohistochemical staining in cartilage tissues obtained from all groups (Table 1). Different patterns of immunopositive cells in each set of specimens were seen. Immunohistochemical staining in chondrocytes from control groups was appreciated mainly in the superficial and middle zone of the cartilage rather than the deep zone, while it was weak/absent in experimental OA cartilage. PACAP immunolabelling was very strong in control groups, without ACLT, ((Figure 3A,B); (ES = ++++; IS = 4)) whereas it was weak/absent in cartilage tissue samples from the OA group (moderate/severe OA), with ACLT ((Figure 3C); (ES = +; IS = 1)). No immunostaining was observed in the negative control (ES = 0; IS = 0) treated with PBS in place of the primary antibody (Figure 3D). The percentage of PACAP-positive cells in OA samples was significantly lower with respect to controls (** *p* < 0.01 *vs.* sham-operated or healthy controls) (Figure 3E). Interobserver agreement, measured as κ coefficient, was 0.90.

**Table 1:** Evaluation of PACAP immunostaining. Intensity of staining (IS) was graded on a scale of 0–4, according to the following assessment: no detectable staining (0), weak staining (1), moderate staining (2), strong staining (3), very strong staining (4). The percentage of PACAP immunopositive cells (Extent Score = ES) was independently evaluated by 3 investigators (2 anatomical morphologists and one histologist) and scored as a percentage of the final number of 100 cells in five categories: <5% (0); 5%–30% (+); 31%–50% (++); 51%–75% (+++), and >75% (++++).

Groups	Intensity of PACAP Immune Staining (IS) and Percentage of PACAP Immuneopositive Cells (Extent Score = ES)
Control rats without ACLT	Very strong immunostaining (ES = ++++; IS = 4)
Control sham-operated rats without ACLT	Very strong immunostaining (ES = ++++; IS = 4)
Experimental rats without ACLT	Weak/absent immunostaining (ES = +; IS = 1)

### 2.4. Effects of ACLT-Induced OA on IL-1β and PACAP Concentration in the SF

**Figure 4:**
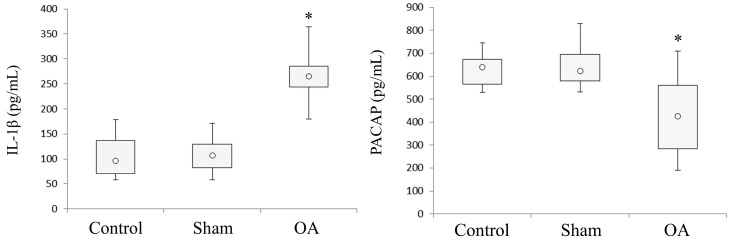
PACAP and IL-1β concentration in the SF collected from the articular cavity of the knee joint from healthy and ACLT-induced OA rats. PACAP and IL-1β concentration in the SF was measured by ELISA method both in controls, sham-operated and OA rat groups. The concentration of PACAP and IL-1β in the box and whisker plots represent the lower and the upper quartile; the open circle in the middle of the box represents the median and the ends of the lines extend to the smallest and largest data point ≤1.5 IQR (interquartile range). (* *p* < 0.05, *vs.* control and sham).

To ascertain whether or not ACLT triggered the local production of the pro-inflammatory cytokine IL-1β but also to investigate whether PACAP levels could be affected by experimental OA, we measured cytokine and peptide levels in the SF using the ELISA method. As depicted in Figure 4, a significant increase of IL-1β concentration in the SF of rats with experimentally induced OA was observed (median 265 pg/mL, range 189–347) in comparison to Controls (median 96 pg/mL, range 61–170) or sham-operated rat (median 107.5 pg/mL, range 61–163) groups. Conversely, PACAP concentrations in the SF were significantly decreased by OA (median 425 pg/mL, range 200–676) as compared to healthy groups (Control median 639.5 pg/mL, range 556–710, Sham median 623.5 pg/mL, range 560–790). These results suggest the existence of an inverse correlation between PACAP and IL-1β concentration in the SF.

### 2.5. Effects of PACAP on IL-1β Induced Chondrocyte Apoptosis

**Figure 5:**
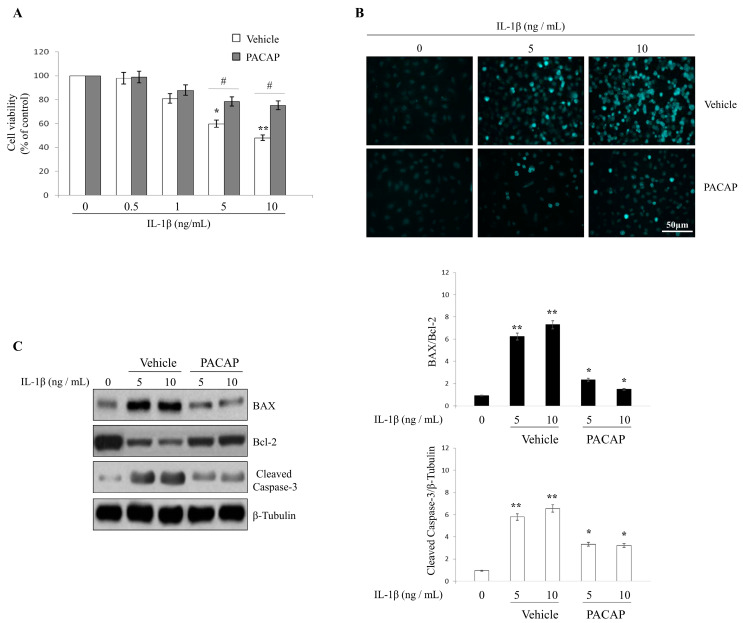
Effects of PACAP on IL-1β induced chondrocyte apoptosis. Chondrocytes were exposed to either vehicle or interleukin 1-β (IL-1β) for 24h at indicated concentrations and cell viability (**A**), DNA damage (**B**) as well as the expression of apoptotic-related proteins (Bcl-2, BAX and Cleaved Caspase-3, respectively) were analyzed as detailed in “Materials and Methods”; (**A**) MTT analyses. Values are expressed as mean optical densities (ODs) ± SEM, obtained from three separate batches of cells, each run in duplicate; (* *p* < 0.05 or ** *p* < 0.01 *vs.* untreated cells; ^#^ *p* < 0.05 *vs.* vehicle); (**B**) Hoechst 33258 nuclear staining. Cells were stained with the fluorescent nuclear dye Hoechst 33258 and viewed at ×40 magnification; Scale bar = 50 μm. At least three randomly selected fields from five independent cultures on cover slips were assessed; (**C**) Western blot analyses of Bcl-2, BAX and Cleaved Caspase-3. Bands were quantified using ImageQuantTL software. BAX/Bcl-2 ratio and Cleaved Caspase-3 normalized values were plotted in the bar graph shown on the right of the representative blots. Quantification of band intensities was performed using the freely available Image J software. Results are reported as average values ± SEM, (* *p* < 0.05 or ** *p* < 0.01 *vs.* untreated cells). Each experiment was performed at least three times using three different batches of cells (*n* = 3).

To investigate whether PACAP prevents the induction of chondrocyte apoptosis probably induced by the inflammatory milieu triggered by OA, we exposed cells to IL-1β and then studied the potential *in vitro* ameliorative effects of PACAP. To pursue this objective, chondrocyte cultures from healthy articular cartilage were isolated through enzymatic digestion and thereafter treated with increasing concentrations of IL-1β. IL-1β concentrations were progressively increased (0, 0.5, 1, 5 and 10 ng/mL, respectively). Thereafter, cells were assayed for viability using the MTT approach 24 h later. As indicated in Figure 5A, the survival rates in IL-1β treated cells were significantly reduced at the highest concentrations tested (* *p* < 0.05 or ** *p* < 0.01 *vs.* untreated cells). Interestingly, PACAP treatment rescued cell viability in comparison to vehicle treated cells (^#^ *p* < 0.05 *vs.* vehicle) (Figure 5A). 

Assessing the presence of morphological signs of nuclear damage and or chromatin fragmentation using the readily accessible Hoechst 33258 staining technique, provided further evidence. Representative images are displayed in Figure 5B. IL-1β treated cells presented the typical morphological features of apoptotic degeneration, which were visibly ameliorated by exogenous PACAP treatment (Figure 5B). In the light of these results, to confirm the apoptotic nature of the degenerative process triggered by IL-1β in chondrocytes, the expression levels of the apoptotic related proteins (Bcl-2, BAX and Cleaved Caspase-3, respectively) were measured by Western blot analyses. As demonstrated in Figure 5C, IL-1β induced a dramatic increase of both the apoptotic executor cleaved caspase-3 (** *p* < 0.01 *vs.* untreated control) and increased the ratio of the pro-apoptotic BAX protein over the anti-apoptotic molecule Bcl-2 in OA (** *p* < 0.01 *vs.* untreated control). Consistent with cell viability and morphological analyses, induction of the apoptosis related proteins by IL-1β treatment was lower in chondrocyte grown in PACAP-containing media compared to vehicles (* *p* < 0.05 *vs.* untreated control), suggesting that PACAP might prevent IL-1β-induced chondrocyte cell death acting as an anti-apoptotic factor.

### 2.6. Effects of PACAP on IL-1β Induced iNOS and COX-2 Expression

**Figure 6:**
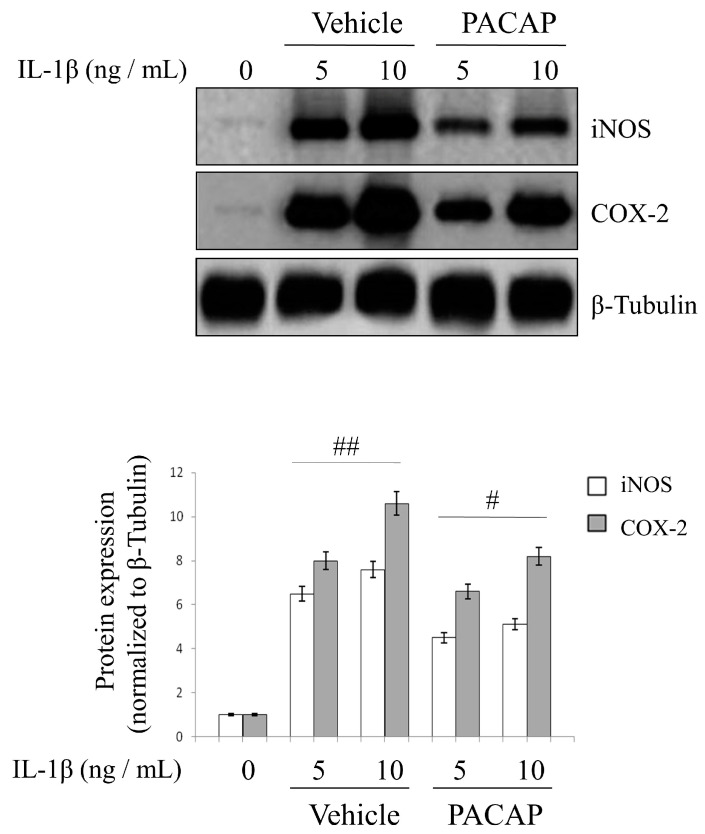
Effects of PACAP on IL-1β induced iNOS and COX-2 expression levels. Chondrocytes were exposed to IL-1β for 24 h at indicated concentrations and changes in iNOS and COX-2 protein levels were measured by Western blot analyses; Bands were quantified using ImageQuantTL software and normalized values were plotted in the histogram shown. Each value represents the mean band densities ± SEM from each group. (^#^ *p* < 0.05 or ^##^ *p* < 0.01 *vs.* vehicle). Experiments were repeated at least three times with similar results.

To determine whether the ameliorative effects of PACAP in chondrocyte cultures were also related to its modulatory activity on other mediators of inflammation, iNOS and COX-2 levels were measured by Western blot analyses using cells cultured and treated as described above. As shown in Figure 6, IL-1β treatment produced a striking increase in both iNOS and COX-2 levels, which was partly but significantly prevented in cells coexposed to PACAP (^#^ *p* < 0.05 or ^##^ *p* < 0.01 *vs.* untreated cells). The association of PACAP treatment and iNOS and COX-2 inhibition suggests that PACAP may be effective in hampering IL-1β-induced inflammatory pathways. 

In the present study we have demonstrated that PACAP levels in the synovial fluid (SF) and in the articular cartilage of the knee joint of rats with ACLT-induced OA are negatively regulated in comparison to healthy controls. Furthermore, using an *in vitro* model of chondrocyte inflammation, we have shown that PACAP is able to significantly diminish IL-1β-induced chondrocyte cell death as well as the expression of inflammatory mediators such as iNOS and COX-2.

Current literature [34,35,36,37] reports that there are currently no available therapies to efficaciously treat or cure OA. More importantly, no evidence has been put forward to indicate the efficacy of an agent or disease-modifying drug that is actually capable of arresting disease progression in an appreciable manner [38,39,40]. Thus, at present, current treatment options are considered only palliative and for the most part are focused on reducing OA associated symptoms, in the attempt to keep affected individuals active. The optimal approach would thus involve the development of a combined strategy focused both on identifying the exact pathogenic mechanisms that contribute to disease onset and progression so that appropriate treatments able to target those aberrantly activated pathways can be developed. Unfortunately, despite many efforts, considering the multifactorial nature of OA disease, this objective remains to be achieved. The aim of the present study was to determine whether levels in SF and cartilage of PACAP, an endogenous bioactive peptide, were affected in rats with experimentally induced OA. PACAP is endowed with many beneficial biological functions in several models of disease [41,42] and may represent a potentially valid candidate for ameliorating certain aspects of this complex disease. In fact, endogenous neuropeptides and hormones released in the immune microenvironment have long been suspected of playing key roles in inflammatory disorders of joints [43]. Juhasz and coauthors [14], reported that PACAPs influence multiple signaling pathways in chondrogenic cells such as the canonical PKA-pathway and the calcineurin–NFAT–BMP axis. The significance of the latter signaling pathway was underlined by the observation that pharmacological inhibition of calcineurin activity diminished cartilage formation and application of PACAPs failed to rescue chondrogenesis in this experimental condition. Chondrogenic cells express molecular elements of PACAP signaling during cartilage differentiation and PACAPs could have a chondrogenesis-promoting effect. Moreover, PACAPs rescued cartilage formation during H_2_O_2_-induced oxidative stress, which raises the possibility of the application of PACAPs during inflammatory joint diseases to protect cartilage or stimulate its regeneration. PACAPs exert their effects via complex signaling mechanisms and it has been proposed that the Ca^2+^-calmodulin dependent phosphoprotein phosphatase calcineurin could be one of the downstream targets of PACAPs in chondrogenic cells [14].

Thus, the original idea of this study came from data present in the literature showing that VIP, a structurally related peptide with high homology to PACAP, has potentially beneficial effects in OA and in other osteoarticular disorders, by down-regulating the inflammatory milieu of the disease [44,45,46]. However, to our knowledge, no data regarding the involvement of PACAP has been published thus far.

In the first part of this paper, we have demonstrated that increased levels of the pro-inflammatory cytokine IL-1β in the SF (provoked by ACLT-induced OA) correlate with a significant decrease in PACAP immunoreactivity in the articular cartilage, as well as its concentration in SF. More specifically, the histomorphometric parameters performed in control groups (without ACLT), confirmed that the animals showed no signs of cartilage degeneration with an intact and normal cartilage structure, whilst in the OA group (with ACLT), cartilage showed clear pathological signatures, evidence of moderate to severe OA, and appearance of horizontal cleavage tears or flaps and deep lesions, all confirmed by Kraus’ modified Mankin score analyses and histopathology OARSI scoring system. These results were further corroborated by histological examinations suggesting that chondrocytes might be unable to maintain their repair activity with subsequent loss of the cartilage tissue. Immunohistochemical analyses showed that PACAP immunostaining was detected in chondrocytes of normal cartilage, mainly in those cells localized in the superficial and middle zone of the cartilage rather than the deep zone. In addition, there was a tendency for a high number of positive chondrocytes in areas of the femoral condyles that are normally exposed to a considerable biomechanical load. Oppositely, PACAP expression was weak or even absent in OA cartilage. This phenomenon was interpreted as a natural down-regulatory mechanism of innate and adaptive immunity, which could also explain the beneficial effects of PACAP in animal models of arthritis [47,48].

**Figure 7:**
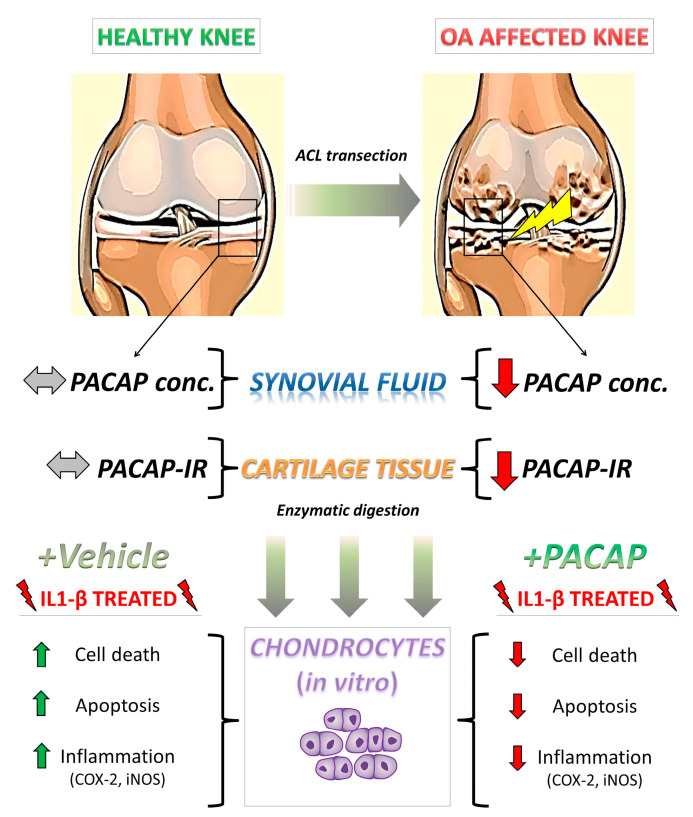
Schematic illustration depicting PACAP regulation in the proposed model of OA and its beneficial effects in isolated chondrocytes after IL-1β insult. Upon anterior cruciate ligament transection (ACLT), rats progressively develop clinical signs of OA, including cartilage deterioration and local inflammatory response [55,56]. In this scenario, PACAP immunoreactivity, which was detectable at high intensities in chondrocytes localized in the outer regions of the healthy cartilage is significantly reduced by experimental induction of OA. Such an event is accompanied by the reduction of PACAP concentration in the synovial fluid and a remarkable increase in intra-articular presence of the pro-inflammatory cytokine IL-1β, suggestive of an active inflammatory process. To assess whether PACAP contributed to prevent chondrocyte death, these cells were isolated through enzymatic digestion and challenged with IL-1β, to partly mimic the inflammatory milieu observed *in vivo*. Results show that PACAP treatment ameliorated most of the detrimental effects of the cytokine, acting both as a chondroprotective and anti-inflammatory molecule. PACAP conc. = PACAP concentration; PACAP-IR = PACAP immunoreactivity.

Chondrocyte apoptosis is known to play a key role in the degeneration and degradation of articular cartilage in cases of OA [49]. Reduced cellularity is a typical feature of the OA cartilage and apoptosis has been proposed as an underlying cause of hypocellularity [50]. Therefore, in the second part of this study we attempted to determine whether adaptive changes in PACAP expression in response to inflammation were implicated in increasing the resistance to cell death stimulated by IL1-β in primary chondrocyte cultures. Data indicated that stimulation with PACAP significantly increased cell viability in cells exposed to IL1-β, and this correlated with attenuation of the apoptotic machinery and release of inflammatory mediators. Although the pleiotropic activities of PACAP have been extensively characterized in different cell cultures and in response to various insults [51], our report is the first to demonstrate *in vitro* a chondroprotective role of PACAP during experimentally induced inflammation (for details on the proposed model please refer to Figure 7). This pro-survival response seems to involve the activation of PAC1/VPAC receptors, which lead to the modulation of intracellular cAMP levels and/or to a cascade of events that ultimately results in the regulation of death and survival genes [52]. However, it is also possible that PACAP might also act indirectly by inducing the release of other growth factors [53,54], although this remains to be ascertained. 

## 3. Experimental Section

### 3.1. Breeding and Housing of Animals

Fifty 6-month-old healthy male Sprague Dawley rats (Charles River Laboratories, Milan, Italy), with an average body weight of 200 ± 40 g were employed in this study. Rats were individually housed in polycarbonate cages (cage dimensions: 10.25"W × 18.75"D × 8"H) during the entire period of the study and were kept at controlled temperature (20–23 °C) and humidity, with free access to water and food chow with a 12 h light/dark cycle. All surgical procedures for anterior cruciate ligament transection were performed in accordance with the method previously described [55,56]. The anterior cruciate ligament transection (ACLT) procedure was made under total anesthesia, 30 mg/kg Zoletil 100 + altadol 5 mg/kg + maintenance mixture of O_2_ and isoflurane 2%–2.5%, (Vibrac, Milan, Italy). Postoperatively, the animals were permitted free cage activity without joint immobilization and were administered an antibiotic, Convenia^®^ 0.1 mL/kg, (Vibrac, Milan, Italy). The 50 animals were distributed in two different groups: 20 rats were used as control group without ACLT, and 30 rats for the OA group with ACLT. The control group consisted of two further subgroups: a naïve control group (10 rats) that did not undergo surgical treatment and a sham-operated control group (10 rats), (*i.e.*, rats that have received exactly the same surgical procedure as the experimental OA group, with the exception that ACL was not transected). The OA group instead consisted of 30 rats that underwent surgical treatment (ACLT) to induce OA. Over the course of the experiments, efforts were made to minimize animal suffering. In particular, rats were monitored for clinical signs of pain (fur appearance, weight loss, lameness, food and water consumption) and their eventual exclusion from the trial was assessed on a daily basis for the entire duration of the experiments. Animals from all groups at 3 months after the surgical procedures were sacrificed by intracardial Pentothal^®^ injection 30–40 mg/kg (Biochemie, Kundl, Austria); under Flurane 2%^®^-narcosis (Abbott Laboratories, Maidenhead, Berks, UK). The pre-operative examinations included physical/macroscopical, photographical and radiographic (X-ray imaging) examination. We selected our sample to represent the range of radiographic OA severity (Kellgren-Lawrence [KL] scores 0 to 4) enriched with knees that showed radiographic worsening over time. According to the Kellgren and Lawrence classification [57], rats had grade 2 (definite osteophytes, definite narrowing of joint space) or grade 3 (moderate multiple osteophytes, definite narrowing of joints space, some sclerosis and possible deformity of bone contour). OA of the knee with definite osteophyte and unimpaired joint space and/or moderate diminution of joint space. Both femurs were explanted, cleaned of soft tissues and the samples were used to perform histomorphometric evaluations. Cartilage tissue was used to perform histological, and immunohistochemical analyses. Isolation of chondrocytes was performed to assay cell viability (MTT), Hoechst staining and Western Blot analyses. Synovial fluid (SF) was collected from both healthy and OA knee joints and used to perform ELISA analyses. All procedures conformed to the guidelines of the Institutional Animal Care and Use Committee (I.A.C.U.C.) of the University of Catania. The experiments were conducted in accordance with the European Community Council Directive (86/609/EEC) and the Italian Animal Protection Law (116/1992).

### 3.2. Histomorphometric Analysis

Femurs were explanted and cleaned of soft tissues as previously described [58]. Histomorphometric analyses were performed on the total number of rats used and specifically on both medial and lateral femoral condyles from all groups (untreated and treated animals). Histomorphometry was performed with image analysis, Kontron KS 300 software (Kontron Electronics, Eching bei München, Germany). Three blinded investigators (3 anatomical morphologists) made the analyses. We assumed that the evaluations were correct if scores among the different investigators were not statistically different. Fifteen fields randomly selected from each section were analyzed. The semi-quantitative histological grading criteria of Kraus’ modified Mankin score [59,60] and histopathology OARSI system [61] were applied.

### 3.3. Histology and Histochemistry

Samples were fixed in 10% neutral buffered-formalin (Bio-Optica, Milan, Italy), following overnight washing and routinely embedded in paraffin as previously described [62]. After wax infiltration the tissue samples were positioned in the cassettes in the same direction. Sections 4–5 μm thick were cut from paraffin blocks using a rotary manual microtome (Leica RM2235, Milan, Italy) and mounted on silane-coated slides (Menzel-Gläser, Braunschweig, Germany) and stored at room temperature. Slides were dewaxed in xylene, hydrated using graded ethanol, and stained as previously described [57] for routine histological evaluation by hematoxylin and eosin (H&E, Bio-Optica, Milan, Italy) staining for general cell identification and for the presence or absence of structural alterations and toluidine blue staining (Fluka, St. Louis, MO, USA) to assess synthesis of sulfated glycosaminoglycan (GAG) containing proteoglycans (assessment was made based on the intensity of staining) [63].

The sections were examined with a Zeiss Axioplan light microscope (Carl Zeiss, Oberkochen, Germany) and photographed with a digital camera (AxioCam MRc5, Carl Zeiss, Oberkochen, Germany).

### 3.4. Immunohistochemistry (IHC)

For immunohistochemical analysis slides were processed as previously described [64]. Briefly, the slides were dewaxed in xylene, hydrated using graded ethanol and incubated for 30 min in 0.3% H_2_O_2_/methanol to quench endogenous peroxidase activity and then rinsed for 20 min with phosphate-buffered saline (PBS; Bio-Optica, Milan, Italy). The sections were then heated (5 min × 3) in capped polypropylene slide-holders containing citrate buffer (10 mM citric acid, 0.05% Tween 20, pH 6.0; Bio-Optica, Milan, Italy), using a microwave oven (750 W) to unmask antigenic sites. The blocking step was performed before application of the primary antibody with 5% bovine serum albumin (BSA, Sigma, Milan, Italy) in PBS for 1 h in a humidified chamber. BSA was used as a blocking agent to prevent non-specific binding of the antibody. Following blocking, the sections were incubated overnight at 4 °C with a rabbit polyclonal antibody developed against PACAP38, working dilution in PBS 1:100 (GTX37576, Gene Tex Inc., Irvine, CA, USA). Immune complexes were then incubated with an anti-rabbit HRP-conjugated secondary antibody and then detected with peroxidase labeled streptavidin, and incubated for 10 min at room temperature (LSAB+ System-HRP, K0690, Dako, Denmark). The immunoreaction was visualized by incubating the sections for 2 min in a 0.1% 3,3'-diaminobenzidine and 0.02% hydrogen peroxide solution (DAB substrate Chromogen System; Dako, Denmark). The sections were lightly counterstained with Mayer’s Hematoxylin (Histolab Products AB, Goteborg, Sweden) mounted in GVA mount (Zymed, Laboratories Inc., San Francisco, CA, USA) and observed with an Axioplan Zeiss light microscope (Carl Zeiss, Oberkochen, Germany) and photographed with a digital camera (AxioCam MRc5, Carl Zeiss, Oberkochen, Germany).

### 3.5. Evaluation of Immunohistochemistry

PACAP-staining was identified as either negative or positive. Immunohistochemical positive staining was defined as the detection of brown chromogen on the edge of the hematoxylin-stained cell nucleus, distributed within the cytoplasm or in the membrane and evaluated as previously described [65]. Staining intensity and the proportion of immunopositive cells were also assessed by light microscopy. Intensity of staining (IS) was graded on a scale of 0–4, according to the following assessment: no detectable staining = 0, weak staining = 1, moderate staining = 2, strong staining = 3, very strong staining = 4. The percentage of immunopositive cells (Extent Score = ES) was independently evaluated by 3 investigators (3 anatomical morphologists) and scored as a percentage of the final number of positive cells over 100 cells using a scaling system based on five categories: <5% (0); 5%–30% (+); 31%–50% (++); 51%–75% (+++), and >75% (++++). Counting was performed under a Zeiss Axioplan light microscope at ×200 magnification. In case of disputes concerning the interpretation, the case was revised to reach a unanimous agreement, as previously described [64]. Digital pictures were taken with a digital camera (Canon, Japan) at 20×, 40× and 63.5× magnifications. Positive and negative controls were assessed to test the specificity of the primary antibodies used in this study. Positive controls consisted of rat brain tissue. Sections treated with PBS in place of the primary antibodies served as negative controls. Positive immunolabeling for antibodies were nuclear/cytoplasmic.

### 3.6. Computerized Morphometric Measurements and Image Analysis

Fifteen fields, randomly selected from each section, were analyzed and the percentage of area stained with PACAP antibody was calculated using image analysis software (AxioVision Release 4.8.2—SP2 Software, Carl Zeiss Microscopy GmbH, Jena, Germany), which quantifies the level of staining intensity of positive immunolabeling in each field. Digital micrographs were taken using the Zeiss Axioplan light microscope (Carl Zeiss, Oberkochen, Germany, using objective lens of magnification ×20, *i.e.*, final magnification ×400) fitted with a digital camera (AxioCam MRc5, Carl Zeiss, Oberkochen, Germany); evaluations were made by three blinded investigators, whose evaluations were assumed to be correct if values were not significantly different. In case of dispute concerning interpretation, the samples were re-evaluated in order to reach a unanimous agreement.

### 3.7. Isolation of Chondrocytes and Culture Conditions

Chondrocytes were isolated from healthy articular rat cartilage using enzymatic digestion. The cartilage pieces were incubated in Dulbecco’s modified eagle’s medium (DMEM, GIBCO, Grand Island, NY, USA) containing 0.2% collagenase (Worthington Biochemical Corporation, Lakewood, NJ, USA) and 5% fetal bovine serum (GIBCO) for 14–16 h at 37 °C and 5% CO_2_. The resulting cell suspension was then filtered through 70 μm nylon filters (Cell Strainer; Falcon, Franklin Lakes, NJ, USA) and washed three times with phosphate buffered saline (PBS) containing 100 U/mL penicillin and 100 μg/mL streptomycin. The number and size of isolated cells was then determined using a Z2 Coulter Counter and Size Analyzer (Beckman Coulter, Inc., Palo Alto, CA, USA). After isolation, chondrocytes were plated onto separate 10 cm tissue culture dishes at a density of 10,000 cells/cm^2^. Cells were incubated at 37 °C and 5% CO_2_ in chondrocyte medium composed of DMEM containing 10% fetal bovine serum, 0.4 mM proline, 50 μg/mL ascorbic acid, 10 mM HEPES, 0.1 mM non-essential amino acid, and 100 U/mL penicillin and 100 μg/mL streptomycin. Culture medium was changed twice weekly. The cells were observed with an Axioplan Zeiss inverted microscope (Germany) and photographed with a digital camera (Canon, Kanagawa, Japan).

### 3.8. Enzyme-Linked Immunosorbent Assay (ELISA)

The concentrations of PACAP and IL-1β in SF were determined using rat PACAP and IL-1β ELISA Kit (MyBioSource, MBS721965 and MBS702717) according to the instructions given by the manufacturer. Optical density was measured at 450 nm using a microplate reader (Bio-Rad, Milan, Italy). Analytical sensitivities of the method were 1.0 pg/mL for PACAP and 15.6 pg/mL for IL-1β.

### 3.9. Western Blot Analysis

Lysates were prepared from subconfluent cells as previously described [66]. Immunoblot analysis was performed by using antibodies listed below: Cleaved Caspase-3 rabbit polyclonal antibody (sc-22171-R, Santa Cruz Biotechnology, Inc., Heidelberg, Germany), Bcl-2 mouse monoclonal antibody (sc-509, Santa Cruz Biotechnology, Inc.), BAX rabbit polyclonal antibody (sc-493, Santa Cruz Biotechnology, Inc.), COX-2 mouse monoclonal antibody (sc-19999, Santa Cruz Biotechnology, Inc.), iNOS rabbit polyclonal antibody (sc-651, Santa Cruz Biotechnology, Inc.) and β-tubulin rabbit polyclonal antibody (sc-9104, Santa Cruz Biotechnology, Inc.) which was used as loading control. All primary antibodies were diluted 1:200, while secondary antibodies (HRP-conjugated goat anti-mouse and anti-rabbit antibodies, Amersham Biosciences) were used at 1:10,000. Blots were developed using enhanced chemiluminescence technique (Amersham Biosciences). No signal was detected when the primary antibody was omitted (data not shown).

### 3.10. Hoechst 33258 Nuclear Staining

The typical morphological features of apoptotic degeneration were analyzed by the use of fluorescence microscopy with the nuclear dye Hoechst 33258. Cells were fixed with a solution of methanol/acetic acid (3:1 *v*/*v*) for 30 min, washed three times in PBS and incubated for 15 min at 37 °C with 0.4 μg/mL Hoechst 33258 dye. After being rinsed in water, cells were visualized for determination of nuclear chromatin morphology with the use of an Axiovert 40 fluorescence microscope (Carl Zeiss Inc., Jena, Germany. Apoptotic cells were recognized on the basis of nuclear condensation and/or fragmented chromatin. Each condition was reproduced in three dishes per experiment. Both apoptotic and normal cells were determined by analyzing at least three different fields per dish in a fixed pattern as previously described [66].

### 3.11. Cell Viability (MTT Assay)

To assess cell viability, we used the cell proliferation kit I (MTT) following manufacturer’s instructions (Roche) but with minor modifications, as detailed in previous studies from our laboratories [67]. Cells were seeded into 96-well plates at a concentration of 1 × 10^4^ cells/well and allowed to adhere for 24 h. Cells were then treated with 0.5, 1, 5 and 10 ng/mL of IL-1β (Sigma-Aldrich, Milan, Italy) for 24 h and, after that, DMEM containing 0.5 mg/mL 3-[4,5-dimethylthiazol-2-yl]-2,5-diphenyltetrazolium bromide (MTT) (Sigma-Aldrich) was added in each well as previously described [68]. Following incubation for 4 h at 37 °C, medium was removed, and 100 μL of DMSO was added. Formazan formed by the cleavage of the yellow tetrazolium salt MTT was measured spectrophotometrically by absorbance change at 550–600 nm using a microplate reader.

### 3.12. Statistical Analysis

Statistical analysis was performed using SPSS software (SPSS^®^ release 16.0, IBM, Chicago, IL, USA). Data were tested for normality with the Kolmogorov-Smirnov test. All variables were normally distributed. Comparisons between two means were tested with the Student’s *t* test, whilst comparison between more than two groups was tested using analysis of variance (ANOVA) and Tukey’s *post-hoc* test. *p*-values of less than 0.05 were considered statistically significant; *p*-values of less than 0.01 were considered highly statistically significant. Data are presented as the mean ± SEM as previously described [13]. Cohen’s κ was applied to measure the agreement between the two observers and averaged to evaluate overall agreement as previously described [69].

## 4. Conclusions

In conclusion, the present paper provides evidence for the expression of PACAP in cartilage and its potential involvement in ameliorating some of the pathophysiological mechanisms involved in OA. The study infers that PACAP beneficial effects against cartilage degeneration may be elicited via two distinct but complementary mechanisms: (1) by reducing chondrocyte apoptosis and (2) by dampening the release of inflammatory mediators. These findings confirmed that PACAP is an endogenous molecule that could be used as a potential chondroprotective agent for the treatment of this harmful condition. Our observations are in accordance with those of Juhasz and coauthors, preparing the ground for a possible therapeutical use of PACAP as protecting agent during joint inflammation and activator of cartilage regeneration during degenerative diseases. These are preliminary data and some clarification of the underlying pathways of PACAP activity are needed to better understand the role of PACAP as a potential chondroprotective agent for the treatment of OA.
