# Correction: Giunta et al. Ameliorative Effects of PACAP against Cartilage Degeneration. Morphological, Immunohistochemical and Biochemical Evidence from *in Vivo* and *in Vitro* Models of Rat Osteoarthritis. *Int. J. Mol. Sci.* 2015, *16*, 5922–5944

**DOI:** 10.3390/ijms252212148

**Published:** 2024-11-12

**Authors:** Salvatore Giunta, Alessandro Castorina, Rubina Marzagalli, Marta Anna Szychlinska, Karin Pichler, Ali Mobasheri, Giuseppe Musumeci

**Affiliations:** 1 Department of Biomedical and Biotechnological Sciences, Human Anatomy and Histology Section, School of Medicine, University of Catania, Via S. Sofia 87, 95123 Catania, Italy; 2 Department of Pediatrics, Clinic for Pediatrics I Medical University of Innsbruck, Anichstr. 35, A-6020 Innsbruck, Austria; 3 The D-BOARD European Consortium for Biomarker Discovery, Department of Veterinary Preclinical Sciences, School of Veterinary Medicine, Faculty of Health and Medical Sciences, University of Surrey, Guildford GU2 7XH, UK; 4 Arthritis Research UK Centre for Sport, Exercise and Osteoarthritis, Arthritis Research UK Pain Centre, Medical Research Council and Arthritis Research UK Centre for Musculoskeletal Ageing Research, University of Nottingham, Queen’s Medical Centre, Nottingham NG7 2UH, UK; 5 Center of Excellence in Genomic Medicine Research (CEGMR), King Fahd Medical Research Center (KFMRC), King AbdulAziz University, Jeddah 21589, Saudi Arabia

In the original publication [1], there was a mistake in Figure 2. The micrograph B representing the sham group was a duplicate of the one representing the control group (micrograph A). The corrected Figure 2 appears below. The authors state that the scientific conclusions are unaffected. This correction was approved by the Academic Editor. The original publication has also been updated.

## Figures and Tables

**Figure 2:**
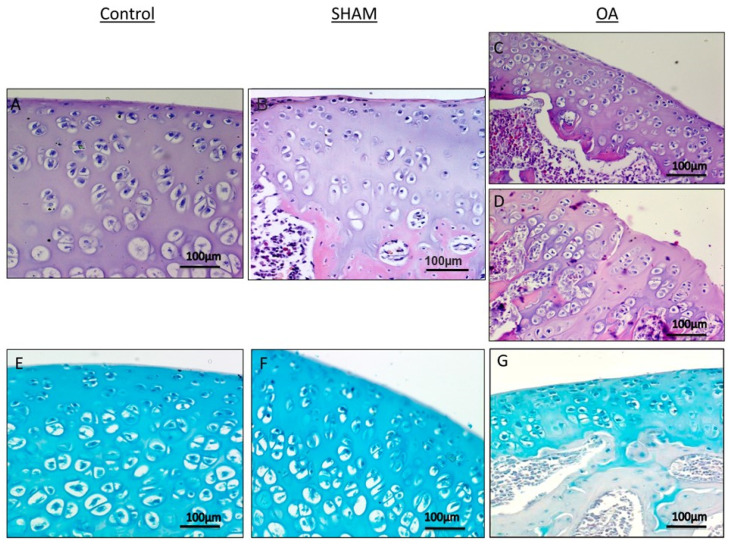
Histological and histochemical evaluation. (**A**,**B**) Histology (H&E staining) demonstrated the absence of structural alterations in control groups (without anterior cruciate ligament transection (ACLT)). In the superficial zone, cells appear flat and small; in the middle and deep zone, cells are organized in columns. Magnification ×20; Scale bars: 100 µm; (**C**) Histology (H&E staining) demonstrated evidence of structural alterations in cartilage with moderate signs of OA (with ACLT). The structural alterations included a reduction of cartilage thickness in the superficial and the middle zones. The tidemark is no longer intact and the subchondral bone shows fibrillation. Magnification ×20; Scale bars: 100 µm; (**D**) Histology (H&E staining) demonstrated signs of structural alterations in severe Osteoarthritis (OA) (with ACLT). Severe OA cartilage shows deep surface clefts, disappearance of cells from the superficial zone, cloning, and a lack of cells in the intermediate and deep zone, which are not arranged in columns. The cartilage layers (superficial zone, middle and deep zone) are completely absent. Magnification ×20; Scale bars: 100 µm; (**E**,**F**) Histochemistry (toluidine blue staining) showed an absence of structural alterations and preserved GAG, in control groups (without ACLT), as indicated by the intense toluidine blue staining. Magnification ×20; Scale bars: 100 µm; (**G**) Histochemistry (toluidine blue staining) demonstrated signs of structural alterations in moderate and severe OA cartilage and loss of proteoglycans as evidenced by poor GAG preservation in the OA group (with ACLT), showing reduced toluidine blue staining. Magnification ×20; Scale bars: 100 µm.
